# Dietary fats as regulators of neutrophil plasticity: an update on molecular mechanisms

**DOI:** 10.1097/MCO.0000000000001055

**Published:** 2024-07-05

**Authors:** Anna Parolini, Lorenzo Da Dalt, Giuseppe Danilo Norata, Andrea Baragetti

**Affiliations:** Department of Pharmacological and Biomolecular Sciences “Rodolfo Paoletti”, University of Milan, Milan, Italy

**Keywords:** cardio-metabolic diseases, dietary fats, innate immunity

## Abstract

**Purpose of review:**

Contemporary guidelines for the prevention of cardio-metabolic diseases focus on the control of dietary fat intake, because of their adverse metabolic effects. Moreover, fats alter innate immune defenses, by eliciting pro-inflammatory epigenetic mechanisms on the long-living hematopoietic cell progenitors which, in the bone marrow, mainly give rise to short-living neutrophils. Nevertheless, the heterogenicity of fats and the complexity of the biology of neutrophils pose challenges in the understanding on how this class of nutrients could contribute to the development of cardio-metabolic diseases via specific molecular mechanisms activating the inflammatory response.

**Recent findings:**

The knowledge on the biology of neutrophils is expanding and there are now different cellular networks orchestrating site-specific reprogramming of these cells to optimize the responses against pathogens. The innate immune competence of neutrophil is altered in response to high fat diet and contributes to the development of metabolic alterations, although the precise mechanisms are still poorly understood.

**Summary:**

Defining the different molecular mechanisms involved in the fat-neutrophil crosstalk will help to reconcile the sparse data about the interaction of dietary fats with neutrophils and to tailor strategies to target neutrophils in the context of cardio-metabolic diseases.

## INTRODUCTION

During evolution mammals adapted to states of nutritional scarcity or to those of abundant availability of food and multiple nutritional sources, by developing the capability of alternating state of fast-refeeding, which probably have guaranteed an evolutionary advantage due to the potential beneficial metabolic effects [1–3]. Over time, however, changes in dietary habits, with an easier access to caloric-dense foods occurred. Today, as a consequence of industrial processing used to refine food flavor and to prolong stability and shelf-life most of the food consumed in daily life is composed by “complex matrices” [4], being poor in healthy nutrients (fibers, vitamins, minerals, and other plant-derived molecules and antioxidants), but enriched in refined sugars and mechanically processed fats (either as saturated, mono- or poly-unsaturated fatty acids), cholesterol, salt, white flour and food additives. Among these nutrients, the content of refined fats represents a critical concern. Indeed, the consumption of fat is more elevated to that of other nutrients (20–40 g of fats are consumed during each meal on average [5]), and meals are commonly served thrice/four times daily [5,6] not only in more developed, but also in emerging low-to-middle income countries. The accumulation of calorie-dense fatty nutrients, together with a hardly balanced energy expenditure (more than a quarter of the global adult population is insufficiently active compared to recommendations [7]) favors the establishment of dominant genetic and epigenetic pathways [8], making the storage of energy as fats in ectopic sites, principally visceral adipose tissue and liver, redundant in some circumstances. Fatty acids (FAs) are released in tissues after the hydrolysis of the triglycerides present in the food matrices that, once absorbed in the intestine, are packed into lipoproteins. As such, there is a delicate balance between the delivery of FAs from triglycerides (TGs) to peripheral cells and their storage in different tissues. In the case of chronic intake of fatty food, the body initially compensates by increasing the storing capacity until a low-grade inflammatory status develops, which, in turn, triggers both insulin-resistance [9,10] and the activation of different immune cell subsets, including the innate immune arm, via an immune-metabolic regulation [11,12].

In search of effective prevention and treatment of the epidemiologically relevant burden of cardio-metabolic diseases, current guidelines are concordant in advising to reduce the dietary intake of saturated FAs, and cholesterol and, to increase the consumption of mono- or polyunsaturated FAs [13]. Whether this approach, which is effective in improving metabolic homeostasis, by reducing insulin resistance and ectopic adiposity, also counteracts the systemic low-grade inflammation is not fully understood. Yet, inconsistent associations between a lower consumption of saturated FAs and a higher intake of mono-/polyunsaturated FAs, with increased plasmatic levels of surrogate markers of low-grade inflammation, have been found in large epidemiological studies [14–16]. Similarly, nutritional interventional trials indicated that, whereas applying the recommendations from guidelines is effective in improving metabolic homeostasis, a beneficial effect in reducing low-grade inflammation is questionable [16]. It is obvious that multiple factors might contribute to these contrasting results. The search of biomarkers, that can be easily quantified is critical and will further contribute to clarify the dichotomy on the pro- or an anti-inflammatory effect of the food matrices. By using multiarray approaches in plasma, we recently identify an association between the consumption of unhealthy fat enriched foods with a set of multiple inflammatory proteins that were clustered into biologically relevant pathways related to the activation and the chemotaxis of innate immune cells, mainly neutrophils [17^▪^]. Being an observational analysis, further studies are required to draw a solid demonstration about a causal effect of dietary fats on the activation of neutrophils. This gap is the consequence of the large bounce of data regarding the reactivity of neutrophils against dietary fats, obtained mainly in vitro. Hence, the conclusion that saturated FAs promote the pro-inflammatory activation of neutrophils while the opposite is true for poly-unsaturated FAs could be too simple. 

**Box 1:**
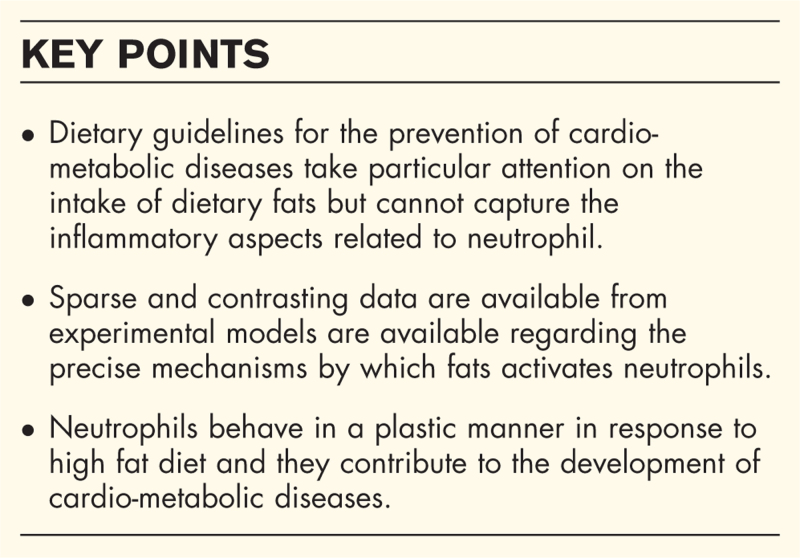
no caption available

### Mechanisms by which dietary fats interact with neutrophils

**FIGURE 1:**
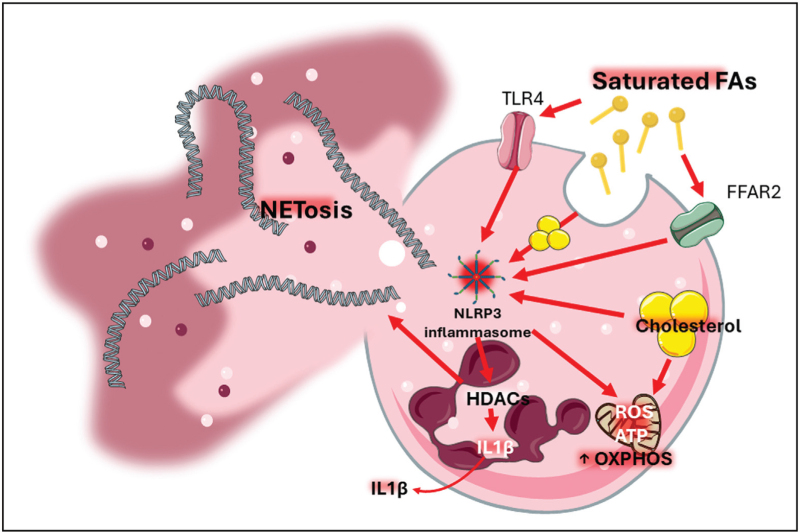
Resume of the mechanisms by which dietary fats interact with neutrophils. The figure resumes the main canonical mechanisms of interaction between dietary fats and the neutrophils that have been described in literature so far. “ATP”, adenosine triphosphate; “FFAR2”, free fatty acid receptor 2 [also termed G-protein coupled receptor 43 (GPR43)]; “NLRP3”, NOD-like receptor protein 3; “OXPHOS”, oxidative phosphorylation; “ROS”, reactive oxygen species; “TLR4”, Toll-like receptor 4; IL1β, interleukin 1 beta.

Short-chain saturated FAs, which originate mostly from the microbial fermentation of fibers complex carbohydrates (that are abundant in vegetables, fruits, legumes, and whole grains [18,19]), in the gut could play different roles in neutrophils (Table 1). Butyrate, for instance, exerts favorable metabolic effects by reducing insulin resistance [20], favoring the transition of monocytes to pro-resolutive anti-inflammatory macrophages, via the inhibition of histone deacetylases (HDACs) [21] and promoting anti-atherosclerotic effects [22]. Yet whether butyrate promotes the pro-resolutive function of neutrophils is unclear. Indeed, some studies indicate that butyrate up-regulates the generation of hydrogen peroxide but, at the same time, reduces that of myeloperoxidase-mediated oxidants (which are critical in killing microorganisms and in inducing tissue injury [23]). Other studies suggest that butyrate impairs the capacity of neutrophils to produce oxidative species in an HDACs-dependent mechanism, and this results into the protection against inflammatory bowel disease [24] (Table 1). Furthermore, other studies suggested that the downregulation of the nicotinamide adenine dinucleotide phosphate, an oxidase complex component required for the generation of reactive oxygen species in neutrophils, impairs the antimicrobicidal activity in the lung [25]. Acetate, by contrast, while promoting glucose intolerance [26] and supporting pro-inflammatory mechanisms on lymphocytes (which promote atherosclerosis [27]), sustains the pro-resolutive activity of neutrophils against *C. difficile* infection, via the interaction with the G-protein coupled free fatty acids receptor, type 2 (FFAR2), that is highly expressed on the membrane of these cells [28,29] (Fig. 1). The activation of the FFAR2 further augments the recruitment of neutrophils to the inflammatory sites, facilitating the activation of the inflammasome system, a complex of cytosolic multiprotein oligomers, that physiologically assembles upon the recognition of either pathogens or inflammatory stimuli, and promotes the release of interleukin 1beta (IL-1β) [30]. IL-1 β, in turn, boosts the expression of anti-inflammatory interleukin 22 (IL-22) by innate lymphoid cells which contributed to the surveillance against pathogen associated invasion of the basolateral membrane of enterocytes [29] (Table 1).

**Table 1:** Effect of dietary fats on metabolism and on the activity of neutrophils

Fatty acid	Effect on metabolism	Effect on neutrophils
Palmitate (16 : 0) C_16_H_32_O_2_	↑ Ectopic adiposity [31] ↑ Insulin resistance [31,38] ↑ Glucose intolerance [55–57]	↑ NETosis [40,41] NLRP3 inflammasome activation and IL-1β release [55] ↑ Oxidative stress [55] ↓ Autophagy [55]
Butyrate (4 : 0) C_4_H_8_O_2_	↓ Insulin resistance [20] ↓ Glucose intolerance [26] Antiatherosclerotic effects [22]	Unclear effects on oxidative species production [23–25] ↓ Myeloperoxidase-mediated oxidants [23]
Acetate C_2_H_4_O_2_	↑ Glucose intolerance [26]	Induction of a pro-resolutive phenotype [28,29] ↑ Neutrophil recruitment [30] NLRP3 inflammasome activation and IL-1β release [30]
Oleate (18 : 1 *n*-9) C_18_H_34_O_2_	↓ Insulin resistance [42–44] ↑ Cardio-metabolic fitness [45,46]	↑ NETosis [40,41] ↓ NLRP3 inflammasome activation [59]
Linoleate (18 : 2 *n*-9,12) C_18_H_32_O_2_	↑ Insulin sensitivity [47]	↑ NETosis [40,41] ↓ NLRP3 inflammasome activation [58]
DHA (22 : 6 *n*-3) C_22_H_32_O_2_	Metabolic improvement [58]	↓ Chemotaxis [48] ↓ NLRP3 inflammasome activation [58]
EPA (20 : 5 *n*-3) C_20_H_30_O_2_	Cardiovascular protection [52] Metabolic improvement [58]	↓ Chemotaxis [48] ↓ NLRP3 inflammasome activation [58] ↓ Membrane rigidity and consequent ↓ inflammatory cytokines production [51]

The table lists the dietary fats for which the effects on metabolism and on the activity of neutrophils are recognized from literature. For each information, the number of the reference that is also present in the main text is reported. “↑” indicates increase while “↓” indicates reduction.

All the types of saturated FAs from diet induce adverse metabolic effects, including ectopic adipose tissue deposition and hepatic insulin resistance [31]. Those favor the development of metabolic syndrome [32], and, in parallel, stimulate a series of pro-inflammatory mechanisms on monocytes [33] and on tissue resident macrophages [34–36]. In spite of these findings, whether and how saturated FAs activate neutrophils, either to produce reactive oxygen species or to release microbicidal and pathogen killing proteins embedded in DNA strands, known as neutrophil extracellular traps (NETs), is yet less clear (Fig. 1). Few data from in-vitro experiments suggest that the length of the alkyl chain might differentially impact the response with the saturated FAs with at least six carbons in alkyl chain promoting the production of radical oxygen specifies while other fatty acids with a similar size [e.g. tricaprin (TC10 : 0), caproic acid (C6 : 0), caprylic acid (C8 : 0) and capric acid (C10 : 0)] do not [37]. Moreover, palmitic acid (C16 : 0), which promotes insulin resistance [38] and atherosclerosis during diabetes [39], could induce the release of NETs in a dose-dependent manner [40,41]. Similarly, oleic acid (C18 : 1) and linoleic acid (C18 : 2) could also trigger the release of NETs in a dose dependent manner, although, differently from palmitic acid, could also exert beneficial metabolic effects (improving insulin resistance in experimental models [42–44], favoring cardio-metabolic fitness in humans [45,46], which in turn improves insulin sensitivity [47]) (Table 1). Historically poly-unsaturated fats, including omega-3 FA, were shown to exert an anti-inflammatory effect by attenuating the chemotactic response of neutrophils and the generation of leukotriene (LT) B4 upon stimulation with calcium ionophores [48]. Nodaway, we recognize that the biochemical interaction of these fats with the cell membrane depends on their biochemistry and profoundly impacts the activation of downstream intracellular signals. Yet, while docosahexaenoic acid (DHA, a 22-carbon alkyl chain omega-3 with 6 unsaturated bonds) increases the rigidity of the membrane and results in a nonuniform stretching related to the presents of cholesterol aggregates (which could be beneficial on neuronal stability and function [49,50]), eicosapentaenoic acid (EPA, a 20-carbon alkyl chain omega-3 with 5 unsaturated bonds) improves the fluidity of the membrane [51], an effect that, in endothelial cells, results into cardiovascular protection [52] and, in neutrophils, reduces the production of inflammatory cytokines ((Table 1).

Despite this evidence, identifying the precise mechanistic interactions of these dietary fats with neutrophils still appears more complex than expected. The downstream effects of the interaction between FAs and the FFARs on the membrane of neutrophils are not completely clear and it is conceivable that some FAs would induce anti-inflammatory responses while others would favor a pro-inflammatory response [53^▪^]. For instance, the activation of the FFARs by omega-3 FA inhibit the signaling of the toll-like receptors (TLRs), via activating peroxisome proliferator-activated receptor gamma (PPAR-gamma) [54]. In neutrophils, TLRs can be activated not only by lipopolysaccharide (LPS) and bacterial pathogens, but also by saturated FAs, favoring the transcription of the pro-IL-1β which, along with the cleavage of pro-caspase 1 into caspase 1 by the NOD-like receptor protein 3 (NRLP3) inflammasome machinery, is released extracellularly as IL-1β to promote inflammation (Fig. 1). Differently from cholesterol that, as crystals, activates the inflammasome by eliciting long-lasting epigenetic mechanisms [55], the activity of FAs on the inflammasome is controversial. Palmitate impairs glucose tolerance, increases insulin resistance *in vivo*[56–58] and activates the NLRP3 inflammasome which fuels mitochondrial oxidative stress and inactivates autophagy [56]. On the other hand, unsaturated FAs, protect from its over-activation. In a murine model of insulin-resistance induced by HFD feeding, the stimulation of bone marrow macrophages with omega-3 FAs suppressed the activation of the NLRP3 inflammasome, thereby inhibiting IL-1β secretion and resulting in improved metabolic alterations *in vivo*[59] (Table 1). Furthermore, oleic acid, reduces the secretion of IL-1b by bone marrow-derived macrophages upon previous stimulation with either LPS or palmitate, supporting that mono-unsaturated FAs can also prevent the over-activation of the NLRP3 inflammasome via the activation of the AMPK pathway [60] (Table 1).

Unfortunately, we do not still have a complete knowledge of the biochemical interaction of FAs with the complex biological systems either on the cell membrane or in the intracellular space in neutrophils. Furthermore, translating data obtained in vitro isolated neutrophils appears more difficult as, *in vivo*, the biology of neutrophils is far complex than previously known. Indeed, a dynamic “plasticity” of these innate immune cells, which can adapt their structure and function during different stages of their half-life (both immediately after their production in the bone marrow (BM), as a consequence of the exposure to the peripheral environment and/or the homing in tissues upon acute inflammatory stimuli, during their disposal in the spleen or when they are called back to BM for re-cycling), is today recognized [61^▪▪^]. We are currently expanding our understanding on the multitude of mechanisms by which these cells relocate in different tissues and reprogram their peripheral function. It is therefore plausible that different types of FAs deriving from diet can promote or derail intra-cellular mechanisms in neutrophils, in a site-specific manner, as a function of the local demands of these cells to fight against pathogens.

### The effect of dietary fats on the plasticity of neutrophils: towards an “immune-metabolic” perspective

**FIGURE 2:**
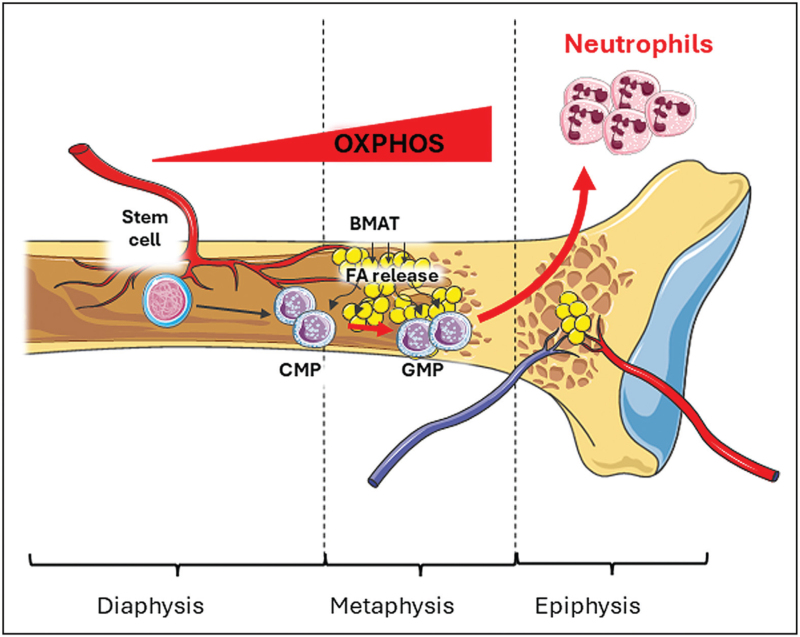
Plastic shape of mature neutrophils from their progenitors in the bone marrow. The figure summarizes the development from hematopoietic stem cells to neutrophils and the changes in cellular metabolic demands that are favored by a different extra-cellular environment over the different regions of the bone marrow. “BMAT”, bone marrow adipose tissue; “CMP”, common monocytoid progenitors; “FA”, fatty acid; “GMP”, granulocytic progenitor cells; “OXPHOS”, oxidative phosphorylation. [CMPs], which give rise to monocytes, and he [GMPs].

So far, cholesterol, but not FAs, has been described as the key activator of the NLRP3 inflammasome in the hematopoietic stem cells, turning on, in an epigenetic manner, the expression of genes that encode for pro-inflammatory and hyper-proliferating signals, including Il-1β [62,63]. Of note, the activation of the inflammasome has been shown also to play a crucial role in the engraftment of hematopoietic stem cells in the BM, favoring the interaction of the G-protein coupled CXCR4 receptor with its constitutive ligand, CXCL12, or Stromal cell derived factor 1 (SDF-1), which reinforce the interaction between integrin α4β1 and its counter receptor VCAM-1 in the stromovascular structure of the niche [64,65]. This mechanism anchors the hematopoietic cell lineages, including the myeloid cell precursors such as the common monocytoid progenitors (CMPs), that give rise to monocytes, and the granulocytic progenitor cells (GMPs), which differentiate to neutrophils [66–69] (Fig. 2). Chronic feeding with a high fat diet (HFD) results into a robust expansion of the granulocytic compartment which acquires a pro-inflammatory phenotype [70], suggesting that some dietary fats can trigger the activation of the inflammasome also at the cell progenitor levels. Moreover, in mice fed a HFD, alarmins like S100A8 and S100A9, produced by neutrophils that infiltrate the visceral adipose tissue, stimulate the release of IL-1β by local macrophages in a TLR4/MyD88/NLRP3 inflammasome-axis dependent manner. IL-1β, in turn, stimulates myelopoiesis in the bone marrow [71]. This observation indicates that the inhibition of TLR4 ligands or the NLRP3-IL-1β signaling axis could be an efficient strategy to reduce inflammation and improve insulin resistance induced by HFD feeding. It is also plausible, although not tested yet, that the HFD could influence the production of NETs, an activity that has been shown to depend on the NLRP3 inflammasome activation as well [72].

FAs reach the BM embedded in lipoproteins. After getting in the medullary microenvironment through the central *nutricia artery* that ramifies in deep of the endosteum (the vascular membrane lining the medullary cavity). Here lipoproteins are hydrolyzed by lipases that are expressed on the membrane of endothelial cells. Of note, these lipases are regulated by several mediators, and, among them, a critical role is played by the Angiopoietin-like protein 3 (Angptl3), which does not only inhibit the activity on the lipoprotein lipases, but also promotes the expansion of the hematopoietic stem and progenitor cells [73]. FAs released by the lipases can be directly recognized by scavenger receptors, including cluster of differentiation 36 (CD36), which have been demonstrated to impact the proliferation of hematopoietic cells. The expression of CD36 on the membrane of hematopoietic stem cells increases during LPS treatment or following *S. typhimurium* infection and is critically involved in the transport of FAs into the mitochondria. This process is mediated by carnitine palmitoyltransferase 1 A (CPT1A), which enables the metabolic switch from glycolysis to FA beta-oxidation thus promoting cell survival [74]. Besides, part of the pool of FAs released after the hydrolysis of TGs can be also stored as bone marrow adipose tissue “BMAT”, which represents the third largest adipose store in the body in physiology and increases its volume in chronic and acute cardio-metabolic conditions [75,76]. BMAT localizes in proximity of the niches, suggesting that a balance between the energy storage and release with lipolysis is crucial for the hematopoietic cells residing nearby [77]. Indeed, the BMAT itself is essential for the hematopoietic expansion, by releasing the stem cell factor in BM [75]. Furthermore, BMAT is spatially organized to provide sufficient energy to sustain the differentiation of all the hematopoietic cells stages [78] (Fig. 2). Yet, an increased density of BMAT is commonly found in the proximal tibia, where it can support the proliferation and the replication of the erythroblasts, the myeloid and the granulocyte lineages [79].

At cellular level, FAs represent a normal key substrate for the mitochondria to fuel energy-yielding cellular mechanisms, including division, proliferation, chemotaxis, and many others. The intracellular availability of FAs favors the activity of essential cellular pathways. While hematopoietic stem cells, physiological rely mostly on an anaerobic metabolism, given that they reside in the hypoxic conditions within the “niche” [80], CMPs and GMPs, by contrast, which move towards more oxygenated and vascularized areas of the BM, need easier access to the FAs substrates to turn on an oxidative, mitochondrial-dependent and more energy-yielding metabolism. Then, the mature neutrophils, which are the downstream lineage of GMPs departing from the BM and released in the vasculature, further undergo an immunometabolic re-shape, becoming short-living cells that, programmed to promptly release their bactericidal arsenal and to eventually undergo suicidal release of NETs to kill pathogens or external invaders, predominantly rely on anaerobic metabolism [81] (Fig. 2). Therefore, while these metabolic changes are well balanced in physiology, it is obvious that an intracellular excess of FAs could drive the overactivation of mitochondria, resulting into an increase of oxidative stress and inflammation [80,82]. These changes in cellular metabolism could be useful when certain immune cells should exert their cytotoxic activity but could be detrimental if it becomes uncontrolled.

We are now aware that the biology of neutrophils is even far more complex. A spectrum of neutrophils entities exists, presenting with different intracellular architecture, abilities to egress from BM niches and to distribute among tissues, and that differ according to site-specific pathophysiological demands. This phenotypic “plasticity”, does not only rely on the anchoring system mediated by the interaction between Cxcl12 and CXCR4 but, also, on the expression of CXCR2, another G-protein coupled receptor on the membrane of neutrophils that, by binding with different affinity to up to eleven chemokines produced by macrophages and epithelial cells in response to inflammatory stimuli, activates downstream signals that regulate the chemotaxis, the phagocytotic potential and the release of NETs, therefore representing a second key orchestrator of the entire life-cycle of the cells [83,84]. CXCR2 and CXCR4 are reciprocal regulator of their membrane expression on these cells not only in physiology, in a circadian manner, but also during acute infections [84,85]. We only recently demonstrated that derailing this interaction, via the use of transgenic mice models where the expression of either CXCR2 or CXCR4 was ablated with the Cre recombinase technology, is also relevant to promote the development of metabolic alterations induced by HFD feeding, including visceral obesity, insulin resistance, liver steatosis and inflammation [86^▪▪^]. Our data thus extend the relevance of a “plastic vision” of neutrophils in the context of chronic cardio-metabolic diseases, although the underlying mechanisms and whether the CXCR2/CXCR4 axis is also essential to regulate the uptake, the utilization of FAs and the immune-metabolic behavior of neutrophils in periphery remains unexplored.

## CONCLUSION

The immune-inflammatory consequences of the cardio-metabolic alterations induced fats-enriched diets pose critical challenges for the development of effective programs for the prevention and treatment of epidemiologically relevant chronic diseases associated with the adherence to fats enriched diets. Moreover, the contemporary guidelines used for the risk assessment only rely on the classical risk factors and cannot consider the underlying inflammatory risk. Therefore, a good proportion of “apparently healthy” subjects who, although exposed to none or few risk factors, daily consume fats enriched meals and do not adhere to healthy lifestyle, are more likely underestimated for their risk of a faster development of cardio-metabolic alterations. The need of a deep understanding of the relationship between nutrition and inflammation is needed and neutrophils appear a core cell compartment for this purpose. However, remarkable, yet unknown, degree of plasticity of neutrophils, coupled with their pervasive role for the maintenance of tissue metabolic homeostasis, draws an “immuno-metabolic” role of these cells in cardio-metabolic diseases. With the currently available therapeutic options, this complex scenario could perhaps complicate the possibility to target neutrophils but, at the same time, might pave the roads towards future interventions to shaping the molecular and behavioral landscape of neutrophils, as a proxy to endorse an efficient and tailored anti-inflammatory approach to reduce the individual risk of developing cardiometabolic diseases.

## Acknowledgements

*This work was supported by: Progetti di Rilevante Interesse Nazionale (PRIN 2022 7KTSAT), Ricerca Finalizzata, Ministry of Health (RF-2019-12370896), Nanokos (European Commission Ref EUROPEAID/173691/DD/ACT/XK), PNRR Missione 4 (Progetto CN3 - National Center for Gene Therapy and Drugs based on RNA Technology), PNRR Missione 4 (Progetto MUSA- Multilayered Urban Sustainability Action to GDN), PNRR Missione 6 (PNRR-MAD-2022-12375913), CARDINNOV, Ministry of Research and University under the umbrella of the Partnership Fostering a European Research Area for Health (ERA4Health) (GA N° 101095426 of the EU Horizon Europe Research and Innovation Programme). Università degli Studi di Milano, Piano Sostegno Ricerca Linea 2 Azione A (PSR2022_DIP_022_AZIONE_A_ABARA). Progetti di Rilevante Interesse Nazionale (PRIN-PNRR 2022 P202294PHK).*

### Financial support and sponsorship

*None to declare.*

### Conflicts of interest

*Authors do not disclose any conflicts of interest which could be relevant for the purpose of the review.*

## REFERENCES AND RECOMMENDED READING

Papers of particular interest, published within the annual period of review, have been highlighted as:

▪ of special interest
▪▪ of outstanding interest
